# Hydrothermal Synthesis of Graphene Quantum Dots Supported on Three-Dimensional Graphene for Supercapacitors

**DOI:** 10.3390/nano9020201

**Published:** 2019-02-04

**Authors:** Peihui Luo, Xiangfeng Guan, Yunlong Yu, Xiaoyan Li, Fengpo Yan

**Affiliations:** Organic Optoelectronics Engineering Research Center of Fujian’s Universities, College of Electronics and Information Science, Fujian Jiangxia University, Fuzhou 350108, China; xfguan@fjjxu.edu.cn (X.G.); ylyu@fjjxu.edu.cn (Y.Y.); xyli@fjjxu.edu.cn (X.L.); fbyan@fjjxu.edu.cn (F.Y.)

**Keywords:** graphene quantum dots, graphene, hydrothermal synthesis, supercapacitors

## Abstract

Incorporation of new functional components into a three-dimensional graphene (3DG) framework improves the performance of supercapacitors based on 3DG as electrodes by tailoring the framework’s structure and properties. In this work, graphene quantum dots (GQDs) were incorporated into 3DG via one-step hydrothermal treatment of GQDs and graphene oxide (GO). By simply adjusting the GQDs/GO feeding ratio by weight, various GQDs/3DG composites were formed. The maximum feeding ratio was 80%, and the prepared composites possessed saturated GQDs loading on the 3DG framework, whereas composites obtained with a GQDs/GO feeding ratio of 40% as electrodes exhibited optimal specific capacitance of 242 F·g^−1^ for supercapacitors, an increase of 22% compared with that of pure 3DG electrodes (198 F·g^−1^). This improved performance was mainly due to better electrical conductivity and larger surface area for GQDs/3DG composites with moderate GQDs content. The fabricated GQDs/3DG composites as electrodes for supercapacitors revealed high electrochemical stability. Their capacitance kept 93% of the initial value after 10,000 charge-discharge cycles.

## 1. Introduction

Three-dimensional graphene (3DG) with a large specific surface area, good electrical conductivity, high electrochemical stability, etc., is ideal electrode material for supercapacitors [1,2,3,4]. Many methods have been developed for synthesizing 3DG structures. Among them, self-assembly of graphene oxide (GO) sheets is widely used under hydrothermal conditions and has advantages such as mass production, no other reducing agent and no need for further purification. This method makes it easy to incorporate various components into the 3DG [5]. Until now, active materials, such as metal nanoparticles [6,7], metallic compounds [8,9,10], polymers [11,12], and carbon materials [13,14], have been successfully decorated onto 3DG, and they exhibited obvious better performance in supercapacitors than in pure 3DG [15,16,17,18].

Graphene quantum dots (GQDs) are single or few-layer graphenes measuring only several nanometers [19]. They are popular for applications in bioimaging and fluorescence sensing due to their particular photoluminescence properties, low toxicity and good biocompatibility [20,21]. Besides, excellent electrical properties, large surface area and abundant active sites are also found in GQDs, making them suitable for utilization in supercapacitors [22,23,24,25,26]. For example, a micro-supercapacitor based on GQDs as electrode materials was prepared via electro-deposition, and it showed superior capability, excellent power response and cycling stability [27]. When adding GQDs to conductive polymers and metal oxides as electrode materials, they also showed obvious improved performance for supercapacitors [28,29,30,31]. Therefore, incorporation of GQDs into 3DG can achieve high performance of 3DG-based supercapacitors through the synergic effects from the two components.

Previously, a few reports related to GQDs/3DG composites for supercapacitors have been published [32,33,34]. One method was electro-deposition of GQDs into 3DG to prepare composites [32]. It was found that the composites with ca. 5% GQDs content showed a higher specific capacitance of 268 F·g^−1^ than that of pure 3DG (only 136 F·g^−1^) at a discharge current density of 1.25 A·g^−1^. The other method was hydrothermal treatment of GO or reduced GO (RGO) with carbon dots (CDs) synthesized by carbonizing small molecules [33,34]. A highest specific capacitance of 338 F·g^−1^ at a current density of 0.5 A·g^−1^ was obtained for formed CDs/3DG composites, and increased by 78% compared with that of 3DG (190 F·g^−1^) [33]. However, electro-deposition for preparing GQDs/3DG composites is time-consuming and prone to cause uneven distribution of GQDs on 3DG. Furthermore, CDs obtained by carbonization of small molecules reveal indistinct structure, which is also unstable, and it is still unclear how the content of GQDs in composites influenced their structures and properties.

Here, GQDs were first prepared via chemical oxidation of carbon fibers. Then, various GQDs/3DG composites were hydrothermally synthesized via adjusting feeding ratio of GQDs and GO by weight. When the GQDs/GO feeding ratio reached 80%, the prepared composites had saturated the GQDs’ content. However, these composites only revealed a specific capacitance of 177 F·g^−1^, which decreased by ca. 11% compared with that of pure 3DG (198 F·g^−1^). A 40% GQDs/GO feeding ratio was optimal for preparing composites applied to supercapacitors. The obtained composites could exhibit a specific capacitance of 242 F·g^−1^, which is more than 22% that of pure 3DG. In a word, incorporation of an appropriated amount of GQDs into 3DG helped the composites in achieve a larger specific surface area and better electrical conductivity, which is beneficial for their application as electrode materials in supercapacitors.

## 2. Methods and Materials

### 2.1. Materials

Graphene oxide aqueous dispersion (2 mg·mL^−1^, sheet size larger than 500 nm) was obtained from Nanjing XFNANO Materials Tech. Co., Ltd (Jiangsu, China). Potassium hydroxide, sodium carbonate, sulfuric acid (98%) and nitric acid (65%) were purchased from Beijing Chem. Plant (Beijing, China). Graphene Quantum Dots were prepared via chemical oxidation of polyacrylonitrile carbon fibers (Zhangjiagang Tariff-free Zone Zhonglibangye International Trade Company, Jiangsu, China) at 100 °C for 24 h according to our previous work [35]. All the reagents were used as received without further purification.

### 2.2. Synthesis of 3DG and GQDs/3DG Hydrogels via One-Step Hydrothermal Method

Fourteen mL GO aqueous dispersion (2 mg·mL^−1^) was sealed in a 22 mL Teflon-lined autoclave and treated at 180 °C for 4 h. The obtained 3DG hydrogel was taken out and immersed in distilled water for the following utilization. To prepare composite hydrogels, 2.8, 5.6, 11.2 or 22.4 mg GQDs were added to the same GO solution as before, and the feeding ratios of GQDs/GO by weight were calculated to be 10%, 20%, 40% and 80%, respectively. After dispersing well, the mixture solution was also sealed in a 22 mL Teflon-lined autoclave and maintained at 180 °C for 4 h. The formed composite hydrogels were taken out and immersed in distilled water for 5 days to remove residual GQDs.

### 2.3. Synthesis of GQDs/3DG Hydrogels via Two-Step Hydrothermal Method

First, a 3DG hydrogel was prepared via one-step hydrothermal method as mentioned above. Then, the 3DG hydrogel monolith was added to 14 mL GQDs aqueous dispersion (0.4 mg·mL^−1^) and sealed in a 22 mL Teflon-lined autoclave. Subsequently, the autoclave was maintained at 180 °C for 4 h again. Finally, the prepared GQDs/3DG composite hydrogel was taken out and immersed in distilled water for 5 days to remove residual GQDs.

### 2.4. Electrochemical Measurement

Electrochemical properties of 3DG and GQDs/3DG composites were measured in three-electrode systems according to previous literature [16]. In our work, 3DG or GQDs/3DG hydrogel pressed on Pt foil was used as the working electrode, and the electrolyte was 1 M KOH aqueous solution. The counter electrode and reference electrode were still RGO hydrogel coated Pt foil and saturated calomel electrode, respectively [36,37]. A model supercapacitor was assembled with the working electrode and counter electrode for the test. The mass loadings of samples for electrochemical measurement were between 2.00 and 4.75 mg·cm^−2^ for each electrode.The specific capacitance was calculated by galvanostatic charge/discharge (GCD) curves using the following formula:

(1) $$C=\frac{I\cdot \Delta t}{m\cdot (\Delta V-IR)}$$

where C is the specific capacitance of unit F·g^−1^; I is the current applied on the electrode of unit A; Δt is the discharge time of unit s; m is the mass of samples loading on each electrode of unit g; ΔV is the potential difference of the GCD process with unit V; and IR represents the voltage drop at the beginning of the discharge process with unit V.

### 2.5. Characterization

Transmission electron microscopy (TEM) images were acquired with a FEI Tecnai G^2^ F30 TEM. The samples were prepared by dispersing 3DG or GQDs/3DG composites in ethanol, then depositing them on copper mesh. Raman spectra were recorded on a HORIBA LabRAM HR Evolution Raman spectrometer with a 514 nm laser beam. Scanning electron microscopy (SEM) images were observed by a ZEISS MERLIN compact ultrahigh resolution Field Emission Scanning Electron Microcopy (FE-SEM). X-ray photoelectron spectroscopy (XPS) was recorded on a Thermo Fisher Scientific ESCALAB 250 Xi XPS spectrometer with Al Kα (1486.6 eV) as the X-ray source and a pass energy of 30 eV. The specific surface area of Brunauer-Emmett-Teller (BET) was measured by a Quantachrome Instruments QUADRASORB evo^TM^ gas sorption surface area and pore size analyzer at ca. 77 K. Electrical conductivity was measured by an RTS-9 4-point probes resistivity measurement system from Guangzhou 4 Probes Tech. Co., Ltd (Guangdong, China). Electrochemical measurements were carried out on a CHI 660D electrochemical workstation under computer control.

## 3. Results and Discussion

**Figure 1:**
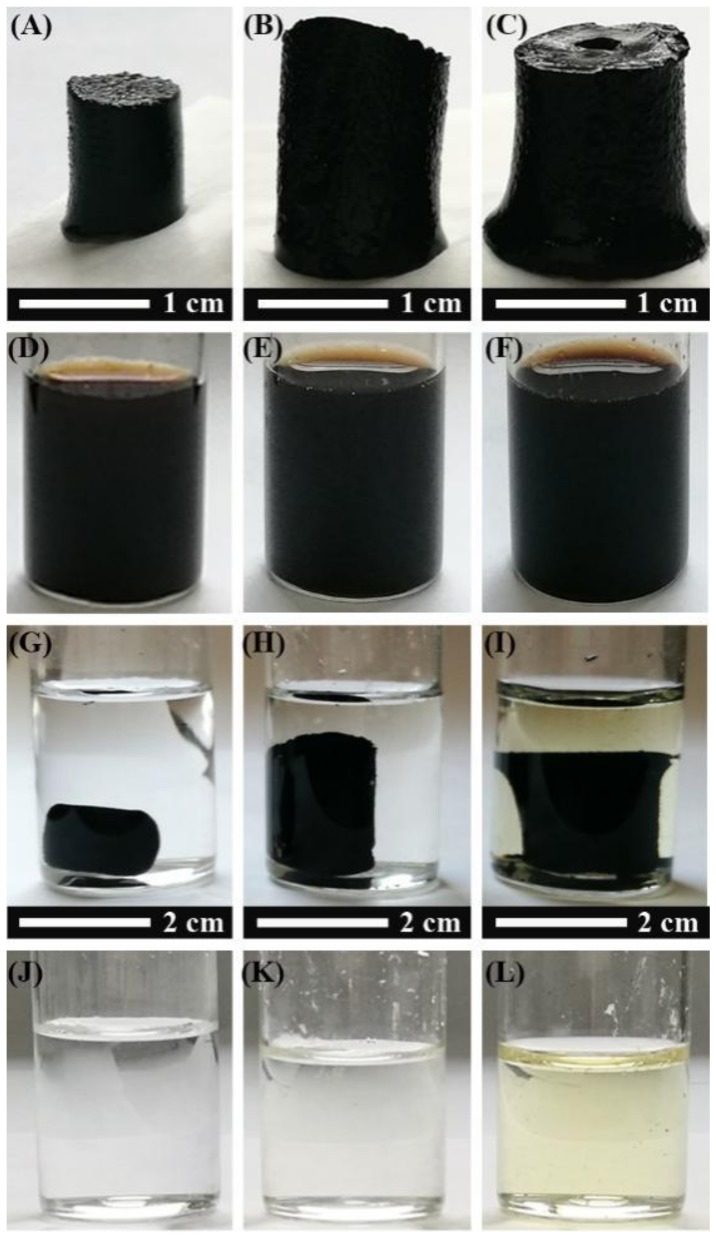
Various photographs for preparing three-dimensional graphene (3DG) (**A**,**D**,**G**,**J**), graphene quantum dots (GQDs)/3DG-40 (**B**,**E**,**H**,**K**) and GQDs/3DG-80 (**C**,**F**,**I**,**L**) via one-step hydrothermal method including synthetic hydrogel, reaction solution before and after hydrothermal treatment, and residual solution after picking out hydrogel.

Three-dimensional graphene was prepared by direct reduction of GO in a hydrothermal environment. In order to obtain GQDs/3DG composites, GQDs raw materials were fabricated by chemically cutting carbon fibers beforehand. The composites were produced via one-step hydrothermal treatment of the mixture of GQDs and GO. The composites prepared using the feeding ratios of GQDs/GO by weight of 10%, 20%, 40% and 80% were denoted as GQDs/3DG-10, -20, -40 and -80, respectively. For comparing with 3DG, typical GQDs/3DG-40 and -80 were selected. Figure 1 shows various optical photographs in the synthetic process of the one-step hydrothermal method. In comparison with pure 3DG hydrogel, GQDs/3DG composite hydrogels had larger sizes, and their cubage increased with more GQDs loading (Figure 1A–C and Figure S1). The used reaction solution for preparing 3DG and its composite hydrogels showed a similar brownish black (Figure 1D–F). After hydrothermal treatment, the residual aqueous solution for preparing 3DG and GQDs/3DG-40 hydrogels was colorless (Figure 1G,H,J,K). However, that of GQDs/3DG-80 appeared a light yellow (Figure 1I,L). It indicates that the GQDs remained in the aqueous solution, which demonstrates saturated GQDs loading for GQDs/3DG-80. Based on the mass of residual GQDs, the effective feeding ratio of GQDs/GO was calculated to be ca. 71% for preparing composites with saturated GQDs loading. Two-step hydrothermal method was also used to prepare GQDs/3DG composites with a GQDs/GO feeding ratio of 20%. It was found that the size of the formed composite hydrogel was similar to that of pure 3DG hydrogel (Figure S2). As a contrast, 3DG hydrogel was added into 14 mL distilled water and hydrothermally treated at 180 °C for 4 h. After the hydrothermal process, the size of 3DG hydrogel was nearly unchanged. Therefore, it was inferred that only a few GQDs could be supported onto the 3DG network via two-step hydrothermal method, which was also confirmed by the residual GQDs aqueous solution with a brown color. Thus, it was difficult to obtain composites with high GQDs content via two-step hydrothermal method. The reason was presumed as follows. In the one-step hydrothermal method, GQDs could interact with GO via chemical reaction by virtue of their various oxygenated groups, π–π and other interactions. However, π–π was the dominant interaction between GQDs and 3DG hydrogel for the two-step hydrothermal method. Also, GQDs with various oxygenated groups on the surface were not easy to interact with RGO via π–π. As a result, only a GQDs/GO feeding ratio lower than 20% was enough to prepare the composites with the saturated GQDs loading.

**Figure 2:**
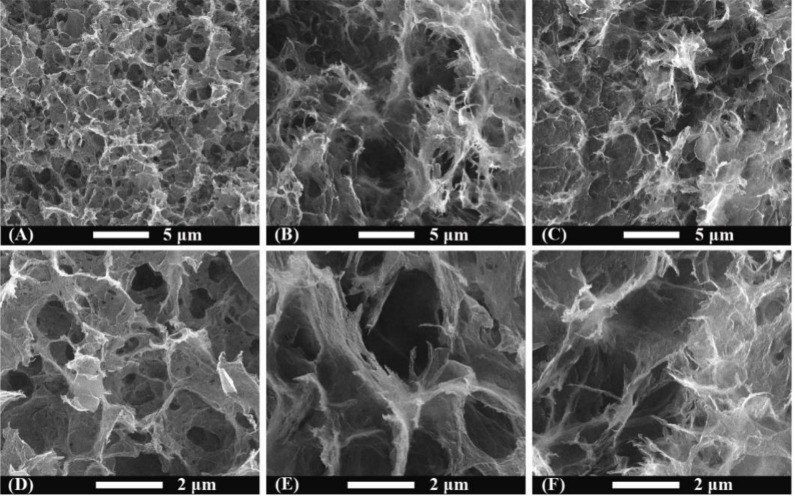
Scanning electron microscopy images of 3DG (**A**,**D**), GQDs/3DG-40 (**B**,**E**), and GQDs/3DG-80 (**C**,**F**) with different magnifications.

The morphologies of samples observed by SEM are shown in Figure 2. Three-dimensional graphene showed various pores of sizes distributed from submicrometer to several micrometers (Figure 2A,D). Compared with 3DG, larger pores and a more fluffy structure were presented for GQDs/3DG-40 (Figure 2B,E), whereas GQDs/3DG-80 showed a more compact structure than pure 3DG (Figure 2C,F). Indeed, the morphologies of 3DG were changed by incorporation of GQDs. A Brunauer-Emmett-Teller test confirmed that GQDs/3DG-40 had a higher specific surface area of 214 m^2^·g^−1^ than 3DG (178 m^2^·g^−1^). On the other hand, GQDs/3DG-80 with a denser structure showed a lower specific surface area, compared with 3DG.

**Figure 3:**
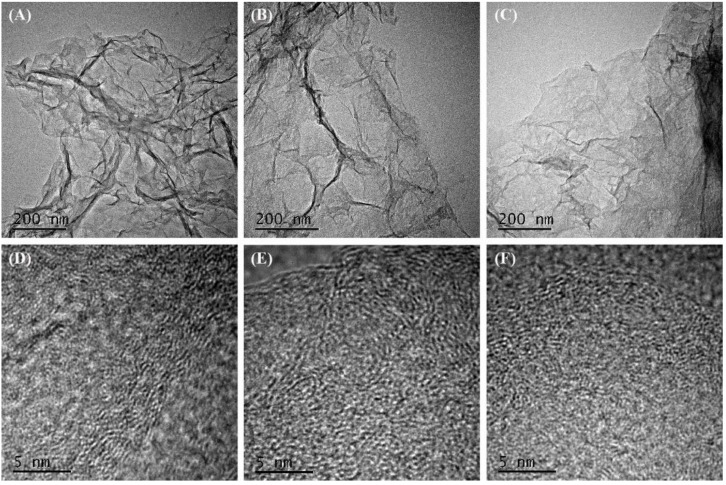
Transmission electron microscopy images of 3DG (**A**,**D**), GQDs/3DG-40 (**B**,**E**), and GQDs/3DG-80 (**C**,**F**) with different magnifications.

Transmission electron microscopy characterization was used to investigate the structure of samples, as shown in Figure 3. From observation of Figure 3A–C, similar graphene sheets with wrinkle structures were presented for three samples. It seemed that there was no obvious difference between GQDs/3DG composites and pure 3DG. This might be ascribed to the similar chemical composition of RGO and GQDs. However, long crystalline stripes longer than 10 nm existed clearly in pure 3DG, as shown by enlargement of the TEM image in Figure 3D. GQDs/3DG composites, by contrast, only revealed a disordered arrangement of crystalline domains, measuring several nanometers (Figure 3E,F), equivalent to the size of GQDs (Figure S3). It indicated that GQDs were successfully supported onto 3DG.

**Figure 4:**
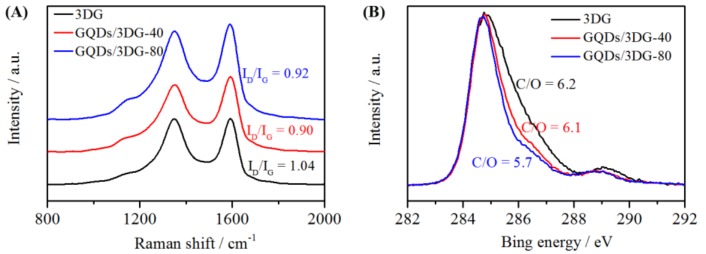
Raman (**A**) and C 1s XPS (**B**) spectra of 3DG, GQDs/3DG-40 and GQDs/3DG-80.

Raman and C 1s XPS spectra are shown in Figure 4. Three samples showed typical D band (at ca. 1355 cm^−1^) and G band (at ca. 1588 cm^−1^), corresponding to disordered and crystalline domains of graphene-related materials, respectively (Figure 4A). The relative intensity ratio of D- and G-band (I_D_/I_G_) indicated the order extent of the samples. Calculated from Raman spectra, I_D_/I_G_ values for 3DG, GQDs/3DG-40 and GQDs/3DG-80 were 1.04, 0.90 and 0.92, respectively. Compared with 3DG, GQDs/3DG composites possessed higher order extent due to the decrease of defects via introduction of GQDs. According to an I_D_/I_G_ value of 0.95 for GQDs after hydrothermal treatment reported by us [35], 0.90 I_D_/I_G_ for GQDs/3DG-40 was the result from the synergistic interaction of hydrothermally treated GQDs and 3DG, whereas I_D_/I_G_ for GQDs/3DG-80 increased slightly, compared with GQDs/3DG-40. This was mainly attributed to excess of GQDs supported on 3DG. X-ray photoelectron spectroscopy measurement revealed that three samples had obvious C=C/C–C related peak at ca. 284.8 eV and C–O/C=O related peaks between 285.5 and 290 eV, respectively (Figure 4B). A few oxygenated groups still existed for these samples, and C/O ratios for 3DG, GQDs-3DG-40 and GQDs/3DG-80 were 6.2, 6.1 and 5.7, respectively. When more GQDs were added, the composites had a higher O content. This was mainly because GQDs, after hydrothermal treatment, still remained in many C=O related oxygenated groups, while most of the oxygen-containing groups were removed from GO in a hydrothermal environment [35].

**Figure 5:**
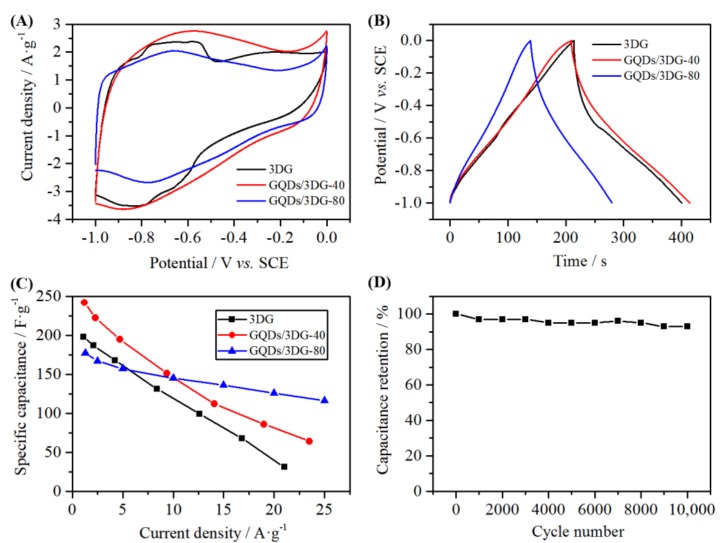
Electrochemical properties of 3DG, GQDs/3DG-40, and GQDs/3DG-80. Cyclic voltammetry (CV) curves at a scan rate of 10 mV s^−1^ (**A**). Galvanostatic charge/discharge curves at current densities of 1.05 A·g^−1^ for 3DG, 1.17 A·g^−1^ for GQDs/3DG-40 and 1.25 A·g^−1^ for GQDs/3DG-80 (**B**). Specific capacitances versus current densities (**C**). Cycling stability at a current density of 24 A·g^−1^ for GQDs/3DG-40 (**D**).

Electrochemical properties were compared in Figure 5. From the cyclic voltammetry (CV) curves at the scan rate of 10 mV s^−1^ (Figure 5A), 3DG displayed obvious redox peaks, which were relevant to residual oxygenated groups after hydrothermal reduction [38]. Redox peaks, however, were obscure, and nearly rectangular CV curves were presented for the composites, indicating that their capacitance was mainly ascribed to electric double layer capacitance. Cyclic voltammetry curves at different scan rates are shown in Figure S4. The coulombic efficiency and specific capacitance of samples were calculated according to galvanostatic charge/discharge (GCD) curve at the current density of ca. 1 A·g^−1^ (Figure 5B). The columbic efficiency value for 3DG, GQDs/3DG-40 and -80 was calculated to be 88%, 98% and 102%, respectively. The GQDs/3DG-40 exhibited a specific capacitance of 242 F·g^−1^ at a current density of 1.17 A·g^−1^, 22% higher than that of pure 3DG (198 F·g^−1^) at a current density of 1.05 A·g^−1^, whereas the specific capacitance for GQDs/3DG-10, -20 and -80 was 204, 213, and 178 F·g^−1^ at current density of 0.80, 1.38 and 1.25 A·g^−1^, respectively. It indicated that GQDs/3DG-40 had the optimal specific capacitance, mainly because GQDs/3DG-40 had larger specific surface area and better electrical conductivity than 3DG and other composites due to moderate GQDs loading. High specific surface area for GQD/3DG-40 was confirmed by BET test and SEM observation. Four-point probes resistivity measurement demonstrated GQD/3DG-40 had better electrical conductivity. The GQDs/3DG-40 showed an electrical conductivity of 0.12 S·m^−1^, higher than that of 3DG (0.06 S·m^−1^) and GQDs/3DG-80 (0.03 S·m^−1^). Electrochemical impedance spectroscopy (EIS) measurement also supported this result (Figure S5). The Nyquist plots showed the impedance in the frequency range of 100 kHz to 0.01 Hz. The x-axis intercept of the Nyquist plot was the equivalent series resistance, and a small semicircle in the high-frequency region was ascribed to charge transfer resistance. Three samples had similar x-axis intercepts and diameters of the semicircle, indicating that they have the same equivalent series resistance and charge transfer resistance. In the low-frequency region, all three samples showed a nearly vertical line, similar to ideal capacitive property [39]. However, their slopes were different. The GQDs/3DG-40 exhibited the largest value, which facilitates electron transport. The curves of the specific capacitances versus current densities are shown in Figure 5C. With increasing current density, specific capacitance decreased rapidly, especially for 3DG and GQDs/3DG-40. The GQDs/3DG-80 showed better rate capability, which might be related to more GQDs functioning as binders for linking graphene sheets. The prepared GQDs/3DG-40 showed excellent cycling stability and exhibited a 93% capacitance retention after 10,000 circles (Figure 5D). Similar composites only retained capacitance lower than 91% of initial value after 5000 circles. Such an improvement for electrochemical stability could be attributed to the combination of high quality GQDs prepared by up-down methods than those obtained via carbonizing small molecules, and strong interaction between GQDs and 3DG via oxygenated groups realized by hydrothermal treatment in our work.

## 4. Conclusions

A convenient one-step hydrothermal method was used to prepare GQD/3DG composites. Various composites can be prepared via simply adjusting the mass ratio of GQDs and GO. The maximum GQDs/GO feeding ratio can be up to ca. 71% for creating a hydrothermal reaction without residual raw materials. The composites formed via using a 40% feeding ratio revealed optimal specific capacitance of 242 F·g^−1^, improved by 22% than that of pure 3DG (198 F·g^−1^). The enhanced performance was mainly attributed to larger specific surface area and better electrical conductivity for GQDs/3DG-40 with moderate GQDs loading. Excess GQDs loading did not benefit for improving structures and properties for 3DG. And the composites exhibited excellent cycling stability with the capacitive retention rate of 93% after 10,000 circles. This might be relevant to strong interaction between GQDs and 3DG via oxygenated groups formed in one-step hydrothermal process, not π−π. Besides, our used GQDs obtained via cutting carbon fibers should possess higher stability than that prepared via carbonizing small molecules.

## Supplementary Materials

The following are available online at http://www.mdpi.com/2079-4991/9/2/201/s1. Figure S1: Photograph of various hydrogels from top view. Hydrogels from left to right are 3DG, GQDs/3DG-10, -40 and -80, respectively. Figure S2: Photographs of different samples in two-step hydrothermal process. The 3DG hydrogel (A). The GQDs/3DG composite hydrogel (B). The used 0.4 mg mL^−1^ GQDs aqueous dispersion (C). Residual GQDs aqueous dispersion (D). Figure S3: Transmission electron microscopy images of GQDs before (A) and after (B) hydrothermal treatment at 180 °C for 4 h. (C) and (D) are the corresponding size distribution. Figure S4: Cyclic voltammetry curves at different scan rate of 3DG (A), GQDs/3DG-40 (B) and GQDs/3DG-80 (C). Figure S5: Nyquist plots of three samples.
