# Supplementary material for: Hydrothermal Synthesis of Graphene Quantum Dots Supported on Three-Dimensional Graphene for Supercapacitors
Source: Nanomaterials (Basel). 2019 Feb 4;9(2):201. doi: 10.3390/nano9020201 (PMC6409901; doi:10.3390/nano9020201)
Supplement: Supplementary file 1 [file nanomaterials-09-00201-s001.pdf]

Supplementary Information

Hydrothermal Synthesis of Graphene Quantum Dots Supported on Three-Dimensional Graphene for Supercapacitors

Peihui Luo *, Xiangfeng Guan, Yunlong Yu, Xiaoyan Li and Fengpo Yan

Organic Optoelectronics Engineering Research Center of Fujian's Universities, College of Electronics and Information Science, Fujian Jiangxia University, Fuzhou 350108, China; xfguan@fjjxu.edu.cn (X.G.); ylyu@fjjxu.edu.cn (Y.Y.); xyli@fjjxu.edu.cn (X.L.); fbyan@fjjxu.edu.cn (F.Y.)

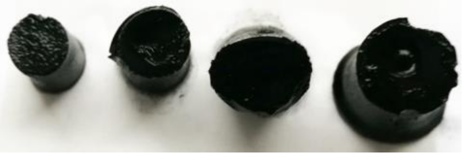

Figure S1. Photograph of various hydrogels from top view. Hydrogels from left to right are 3DG, GQDs/3DG-10, -40 and -80, respectively.

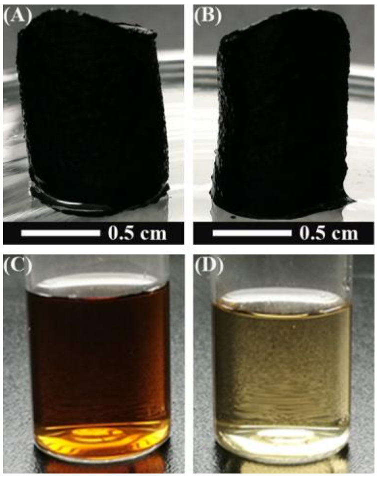

Figure S2. Photographs of different samples in two-step hydrothermal process. 3DG hydrogel (A). GQDs/3DG composite hydrogel (B). The used 0.4 mg mL^{-1} GQDs aqueous dispersion (C). Residual GQDs aqueous dispersion (D).

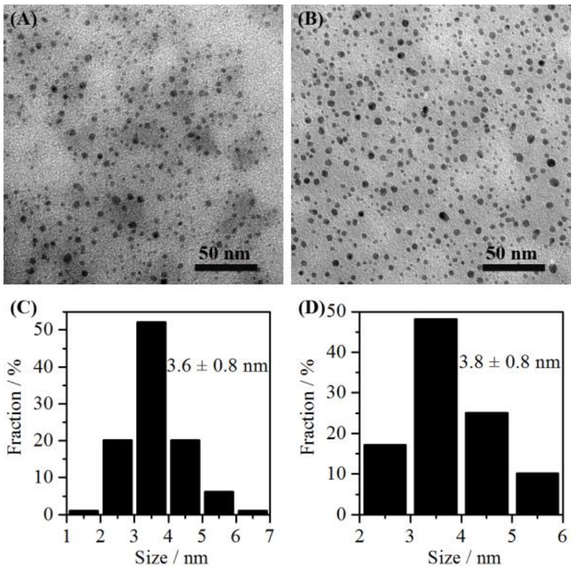

Figure S3. TEM images of GQDs before (A) and after (B) hydrothermal treatment at 180 °C for 4 h. (C) and (D) are the corresponding size distribution, respecitvely.

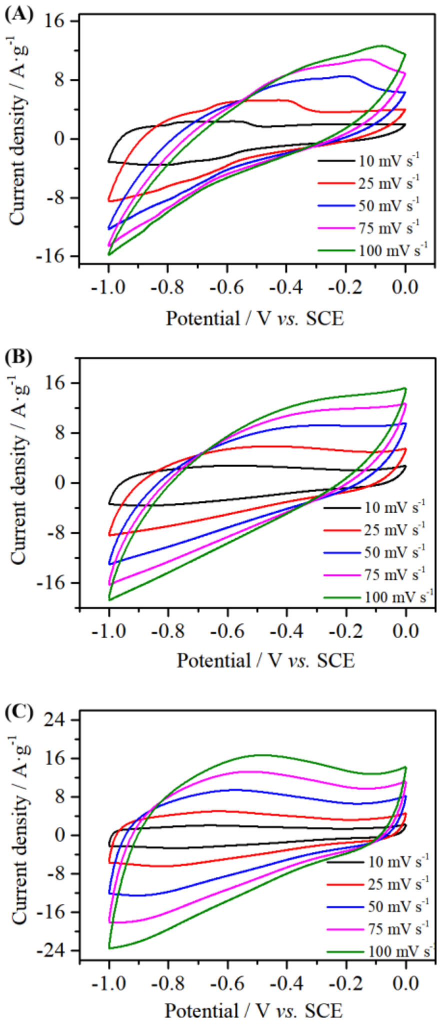

Figure S4. CV curves at different scan rate of 3DG (A), GQDs/3DG-40 (B) and GQDs/3DG-80 (C).

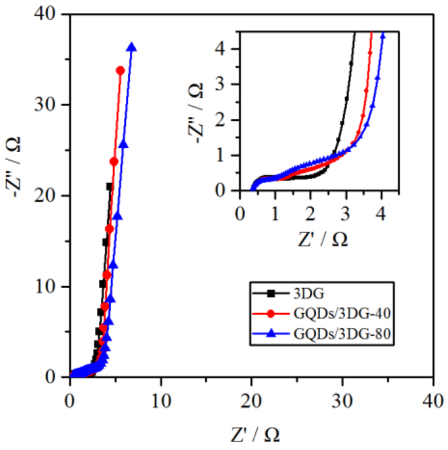

Figure S5. Nyquist plots of three samples
